# Co-culturing neural and bone mesenchymal stem cells in photosensitive hydrogel enhances spinal cord injury repair

**DOI:** 10.3389/fbioe.2024.1431420

**Published:** 2024-12-16

**Authors:** Jianzhong Bai, Guoping Liu, Yang Gao, Xishan Zhang, Guoqi Niu, Hongtao Zhang

**Affiliations:** ^1^ Department of Orthopedics, The First Affiliated Hospital of Soochow University, Soochow University, Suzhou, Jiangsu, China; ^2^ Department of Orthopedics, The Second Affiliated Hospital of Shandong First Medical University, Tai’an, China; ^3^ Department of Spine Surgery, The Second Affiliated Hospital, Hengyang Medical School, University of South China, Hengyang, Hunan, China; ^4^ Department of Orthopedics, The Second Affiliated Hospital of Bengbu Medical University, Bengbu, Anhui, China

**Keywords:** neural stem cell, bone mesenchymal stem cells, co-culture, photocrosslinkable gelatin hydrogel, spinal cord injury

## Abstract

In mammalian species, neural tissues cannot regenerate following severe spinal cord injury (SCI), for which stem cell transplantation is a promising treatment. Neural stem cells (NSCs) have the potential to repair SCI; however, in unfavourable microenvironments, transplanted NSCs mainly differentiate into astrocytes rather than neurons. In contrast, bone mesenchymal stem cells (BMSCs) promote the differentiation of NSCs into neurons and regulate inflammatory responses. Owing to their easily controllable mechanical properties and similarities to neural tissue, gelatin methacrylate (GelMA) hydrogels offer remarkable cell biocompatibility and regulate the differentiation of NSCs. Therefore, in this study, we propose co-culturing NSCs and BMSCs within low-modulus GelMA hydrogel scaffolds to promote regeneration following SCI. *In vitro* comparisons revealed that the viability, proliferation, migration, and neuron differentiation capacity of cells in these low-modulus scaffolds exhibit substantially superior performance compared to those in high-modulus hydrogel scaffolds. To the best of our knowledge, this study is the first to report that NSCs/BMSCs co-culture implants can remarkably enhance motor function recovery in SCI rats, reduce the area of spinal cord cavities, stimulate neuron regeneration, and suppress scar tissue formation. Thus, this hydrogel system loaded with co-cultured cells represents a promising therapeutic approach for SCI repair.

## 1 Introduction

Spinal cord injury (SCI) can lead to persistent motor and sensory functional impairments, and even paralysis in affected individuals (Fan et al., 2022), which reduces the quality of life and increases economic burden (Kumar et al., 2018). The inhibitory microenvironment, inflammatory response, and scar tissue formed at the site of SCI can inhibit the regeneration of spinal axons and the recovery of relevant neuronal functions (Orr and Gensel, 2018; Sun et al., 2019a). Therapeutic interventions following SCI remain a formidable challenge in clinical settings, whereas stem cell transplantation offers a potential treatment for post-SCI repair (Tran et al., 2018).

Neural stem cells (NSCs) are multipotent, self-renewing cells with the potential to differentiate into three types of neural cells, namely, neurons, oligodendrocytes, and astrocytes (Liu et al., 2023). NSCs can promote neural repair by enhancing connections between synapses of neurons at the injury site through the secretion of various neurotrophic factors. In addition, NSCs possess anti-inflammatory properties and can inhibit scar tissue formation (Guo et al., 2022). Neurons are crucial for the reconstruction of neural networks and the restoration of functional movement. However, in the absence of effective intervention, most NSCs transplanted to the injury site tend to differentiate into astrocytes rather than neurons, leading to the formation of glial scars a few weeks post injury. This results in the separation of the injured segments and failure of axonal regeneration (Vieira et al., 2018; Xue et al., 2021). Therefore, it is essential to establish a neurogenic niche that can facilitate exogenous NSCs to overcome their strong inherent tendency towards glial differentiation. The application of mesenchymal stem cells (MSCs) could potentially provide a therapeutic strategy for enhancing regeneration post SCI. MSCs possess the ability to influence the differentiation of NSCs while concurrently promoting their growth and proliferation (Oh et al., 2011; Haragopal et al., 2015). Studies have shown that even when basic fibroblast growth factor (bFGF) and epidermal growth factor (EGF) are removed from the culture medium, co-culture with MSCs can maintain NSC stemness (Stewart et al., 2017). Additionally, NSCs exert an impact on the neuronal differentiation of MSCs through the secretion of brain-derived neurotrophic factor and nerve growth factor, and the transcriptional characteristics of these two types of stem cells also undergo modifications during co-culture (Robinson et al., 2011).

Bone marrow mesenchymal stem cells (BMSCs) express paracrine factors to promote axon and neuronal regeneration and limit further damage to the spinal cord by inhibiting the inflammatory response (Mukhamedshina et al., 2019; Maldonado-Lasuncion et al., 2021; Zhai et al., 2021). Co-culturing human NSCs (hNSCs) with human BMSCs can greatly boost hNSC proliferation while improving hNSC stemness and adherence through the Notch-1 signalling pathway (Haragopal et al., 2015). Stem cell transplantation is an effective treatment in SCI models; however, the differentiation ability of these cells can be compromised by the inflammatory response and inhibitory microenvironment following SCI. Furthermore, the survival of cells at the injury site is markedly low, thereby impeding the restoration of the continuity of neurons in spinal cord tissue. These factors result in the limited effectiveness of conventional stem cell injection methods in treating SCI (Fan et al., 2022).

Recently, biological scaffolds have been developed for SCI treatment. By implanting the biological scaffold into the lesion cavity of SCI, it is possible to restore the continuity of the spinal cord tissue. Nevertheless, most biological scaffolds lack adequate biocompatibility, degradability, and mechanical properties, and cannot simulate the physiological microenvironment of spinal cord tissue. This poses a challenge to the promotion of SCI repair (Song et al., 2019). Gelatin methacrylate (GelMA) is a photosensitive hydrogel prepared by cross-linking gelatin and methacrylic anhydride under a photoinitiator (Velasco-Rodriguez et al., 2021). Its precursor, gelatin, is a natural material, mainly formed by a denatured collagen (Vigata et al., 2020; Kim et al., 2022). Gelatin solution also possesses unique low-temperature gel properties and can form crosslinked hydrogels with other materials (Sharifi et al., 2021). Nevertheless, its application is limited by the uncontrollable degradation rate and poor mechanical properties (Sharifi et al., 2021). In contrast, GelMA hydrogel synthesised from methacrylic anhydride and gelatin has superior mechanical properties and biocompatibility. By altering the GelMA hydrogel concentration, initiator concentration, and photopolymerisation duration, the mechanical properties of the hydrogel can be changed to mimic the physiological features of the spinal cord (Chansoria et al., 2021). Such modulus regulation not only facilitates the adhesion, diffusion, and proliferation of different types of cells in the hydrogel scaffold but also promotes the neuronal differentiation of NSCs (Beck et al., 2016; Fan et al., 2018). Therefore, GelMA hydrogel can offer the microenvironment required for co-cultured cell growth and regulate the differentiation of loaded stem cells.

**FIGURE 1:**
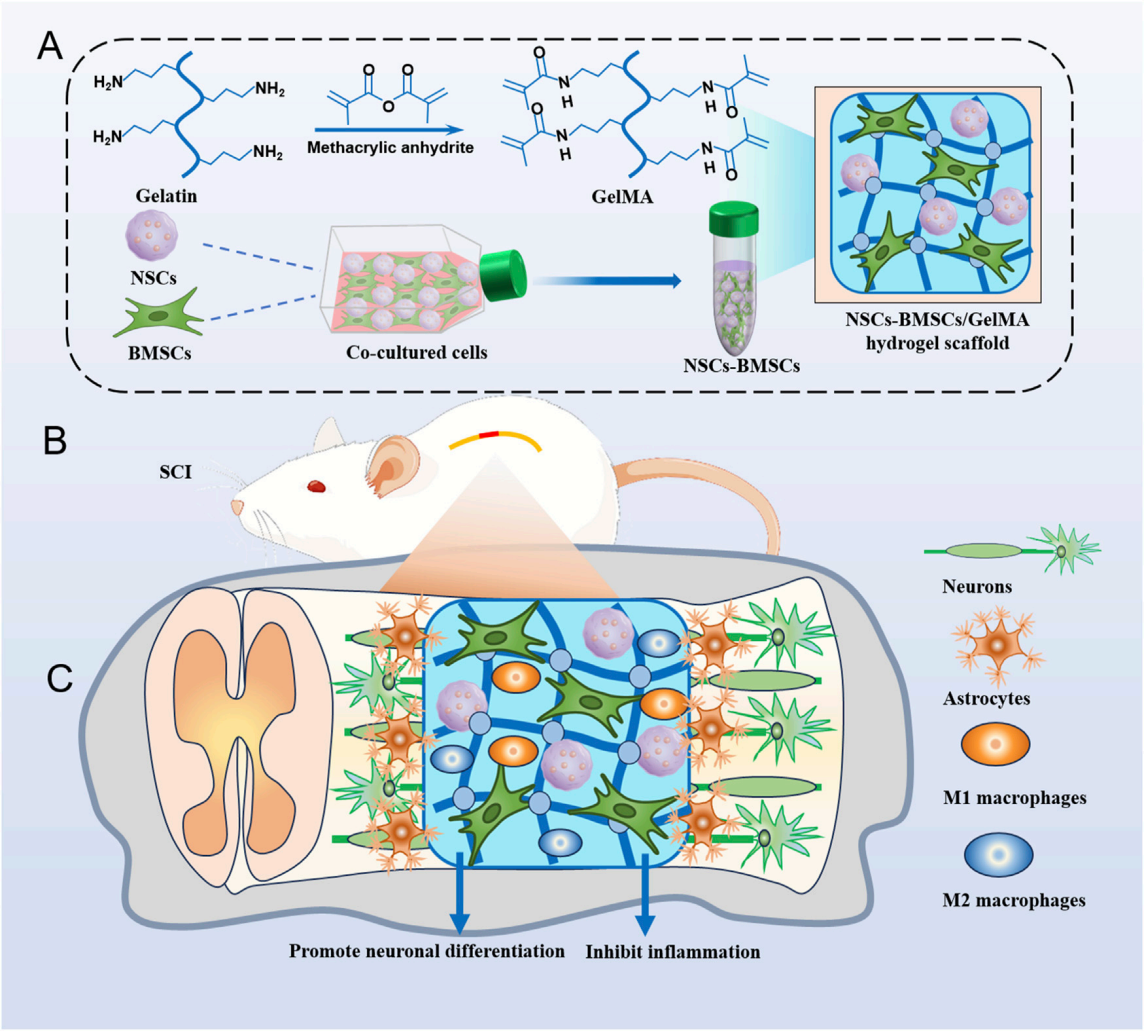
Hydrogel scaffolds loaded with co-cultured cells promote nerve regeneration after SCI. **(A)** Illustrates the preparation of GelMA hydrogel and the encapsulation process of co-cultured cells NSCs and BMSCs. **(B)** Surgical procedure of cell scaffold implantation in a rat spinal cord hemisection model. **(C)** Cell scaffolds reduce inflammation and promote tissue remodeling and nerve regeneration.

Inspired by these studies, we modulated the concentration of hydrogel to mimic the structure and mechanical properties of spinal cord tissue and modulated the neuronal differentiation of NSCs by delivering NSCs and BMSCs (Figure 1). We investigated the effects of three different concentrations of GelMA hydrogel on the growth, proliferation, migration, and neuronal differentiation of co-cultured cells *in vitro*. In a rat hemisected SCI model, we implanted a cellular scaffold and observed the area of the spinal cord cavity, neural regeneration, and the restoration of motor function. Our research offers proof of concept for a biomimetic hydrogel scaffold of neural tissue loaded with co-cultured cells to alleviate secondary damage following SCI and promote neural regeneration.

## 2 Materials and methods

### 2.1 Hydrogel scaffold preparation

**FIGURE 2:**
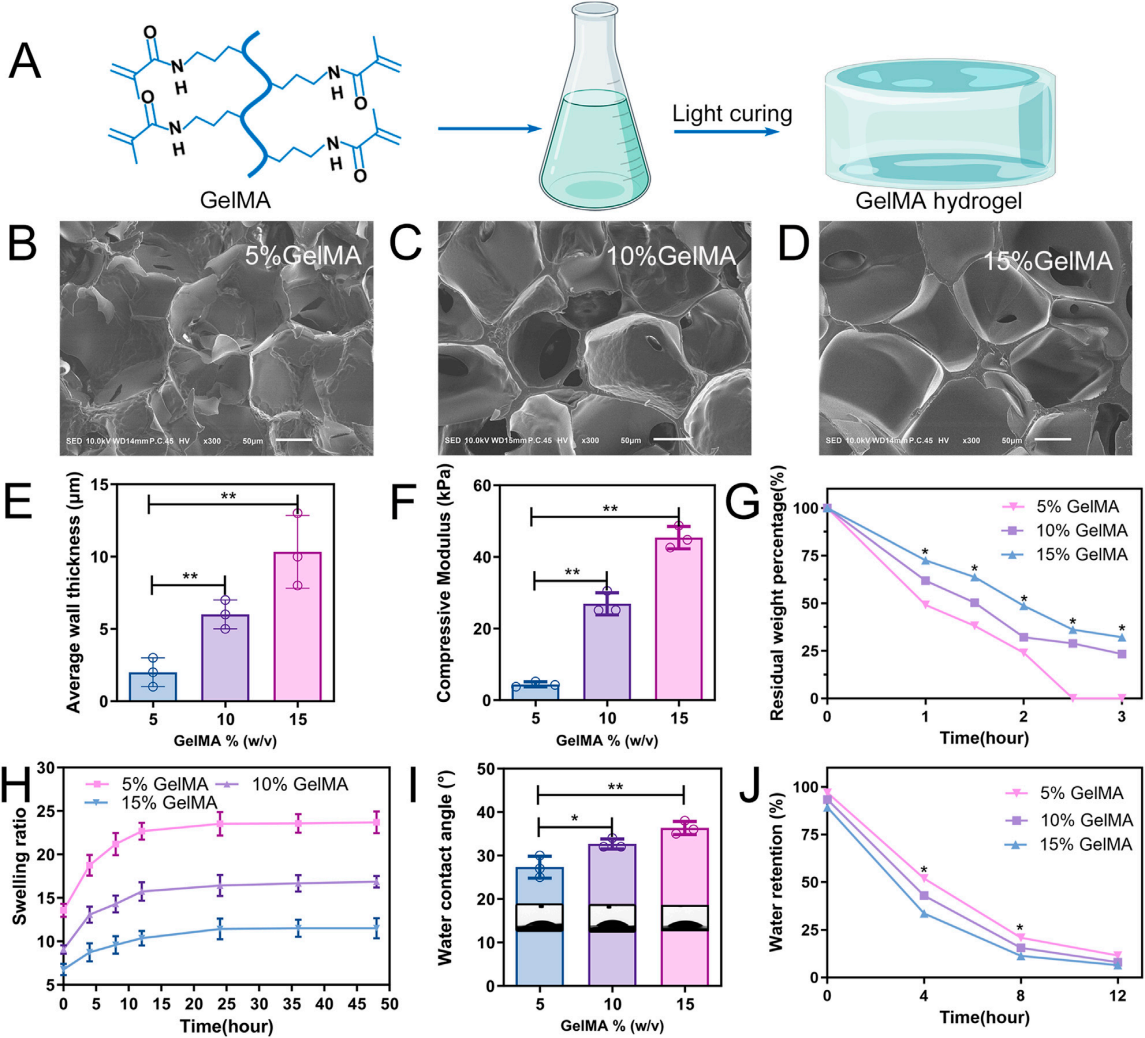
Characterization of GelMA hydrogels. **(A)** Schematic diagram of the photocuring GelMA hydrogel. **(B–D)** Representative SEM images of three hydrogel scaffolds. **(E)** Average pore wall thickness of GelMA hydrogels with different concentrations. **(F)** Compression modulus of hydrogel. **(G)** Mass retention during hydrogel degradation. **(H)** Swelling ratio of the hydrogel. **(I)** Water contact angle analysis. **(J)** Water retention curve of the hydrogel (n = 3; *p < 0.05, **p < 0.01).

A total of 10 g gelatin (EFL, Suzhou, China) was added to 100 mL PBS solution and stirred in a water bath at 60°C for 1 h until completely dissolved, following which methacrylate (EFL, Suzhou, China) was slowly added. After the reaction, the mixed solution was added into a 12–14 kDa regenerated cellulose dialysis bag (Shanghai Bohu Biology, Shanghai, China) for dialysis for 1 week to remove unreacted methacrylate and by-products, then centrifuged to obtain the supernatant, and stored at −80°C and freeze-dried for later use. GelMA was prepared at three concentrations (5%, 10%, and 15%; w/v) in deionized water. The hydrogel was cured by cross-linking reaction with 0.05% (w/v) water-soluble photoinitiator lithium aryl phosphonate (LAP) under ultraviolet (UV) light at 365 nm (Figure 2A).

### 2.2 Characterisation of the physical properties of hydrogels

#### 2.2.1 Morphology of the hydrogel

The three concentrations of GelMA samples were freeze-dried at −80°C and broken from the middle, fixed on the sample table, and sputter coated with gold (SC7620, Quorum Technologies, UK) for 90 s. The porous structure of the truncated surface of the hydrogel was observed using scanning electron microscope (SEM; Hitachi, Kyoto, Japan). ImageJ software was used to measure the average pore wall thickness of the samples.

#### 2.2.2 Compression modulus of the hydrogel

Hydrogel scaffolds of the three concentrations were made into discs with an 8 mm diameter and a 4 mm height prior to testing. The scaffolds were then immersed in PBS overnight at 37°C. Subsequently, the compression modulus was measured on a mechanical tester (Shanghai Heng Yi, China) at a strain rate of 20%.

#### 2.2.3 Biodegradation of hydrogels

The hydrogel scaffolds were weighed as W_0_ under drying and subsequently placed in 2 U/mL collagenase type II solution (Sigma, Germany) at room temperature (25°C), removed at the set time points, freeze-dried, weighed, and recorded as W_t_. Its degradation rate (WD) was computed at various time intervals using the following equation: WD = W_t_/W_0_ × 100%.

#### 2.2.4 Swelling assay

The hydrogels were prepared as discs after photopolymerisation as described for mechanical testing, and the samples were incubated in PBS at 37°C. At each interval, the hydrogel was removed, and the swelling weight (W_s_) of the hydrogel was recorded. The samples were then freeze-dried and the dry weight (W_d_) was recorded again. The ratio of W_s_ to W_d_ was used to calculate the swelling ratio of the hydrogel.

#### 2.2.5 Wettability analysis

To assess the scaffold’s wettability, the hydrogel scaffold was mounted to the worktable, and the droplet shape analysis equipment was used to analyze the hydrogel’s water contact angle at 25°C.

#### 2.2.6 Water retention properties of hydrogels

After being freeze-dried and weighed as W_0_, all hydrogels were submerged in PBS for 24 h at 37°C to allow for water absorption and swelling before being weighed as W_t_. The hydrogel was then placed at 25°C and weighed after removing water from the surface at different time points. The following equation was used to compute the water retention capacity (W_R_) at various time intervals: W_R_ = (W_t_–W_0_)/W_0_ × 100%.

### 2.3 *In vitro* study

#### 2.3.1 Isolation and culture of NSCs

Spinal cord-derived NSCs were extracted from Sprague–Dawley (SD) rat embryos at 14 days of gestation as previously described (Hu et al., 2012). Briefly, spinal cord tissue was cut as finely as possible and treated in digestive enzymes at 37°C for 10 min. The sample was then filtered through a 70 μm filter and centrifuged to obtain a cell suspension, which was cultured at a cell density of 5 × 10^5^/mL in NSC complete medium. The NSC complete medium consists of serum-free DMEM/F12 (Gibco, United States) with 20 ng/mL basic fibroblast growth factor (bFGF) (Peprotech, United States), 20 ng/mL epidermal growth factor (EGF) (Peprotech), and 2% B27 (Gibco). Floating neurospheres were collected from the proliferation media after 3–5 days of growth.

#### 2.3.2 Isolation and culture of BMSCs

Under sterile conditions, BMSCs were isolated from the tibia of 6-week-old male SD rats that were expressing green fluorescent protein (GFP). The donor rats were sacrificed via cervical dislocation, and the attached muscles on the tibia were removed and washed with PBS. After the two ends of the bone were severed, a DMEM/F12 medium was used to clean out the medullary cavity. The collected bone marrow tissue was filtered through a 100 μm nylon mesh. The obtained BMSCs were seeded at a cell density of 5 × 10^6^/mL in MEM/F12 medium with 10% foetal bovine serum (FBS) (Hyclone) and 100 U/mL penicillin and streptomycin (Invitrogen, United States). The third-generation BMSCs were employed in the experiments, and the medium was replaced every 3 days.

#### 2.3.3 Identification of NSCs and BMSCs

Suspended neurospheres were fixed with 4% paraformaldehyde (v/v; Sigma), washed with PBS, and blocked with 10% donkey serum prepared with 0.5% Triton X-100 (Invitrogen) for 2.5 h. The blocking solution was removed by suction, and the primary antibody (Nestin) was added and placed at 4°C overnight. After the cells had been fully treated with the primary antibody, they were rinsed three times with PBS before being added to a diluted secondary antibody (goat anti-rabbit IgG that had been conjugated to Alexa Fluor 488) and incubated for 1 h at 25°C in the dark. After washing, DAPI stain (Invitrogen) was added for counterstaining and observed under an inverted microscope. Supplementary Table S1 includes a list of the primary and secondary antibodies used.

The BMSC phenotype was identified using flow cytometry. To create a solution of 1 × 10^6^ single cells per mL, 0.25% trypsin solution (EDTA-free, Gibco; Cells were digested for 2 min) was used to digest the third-generation BMSCs. Five test tubes were taken, and after addition of 100 μL of single-cell suspension to each test tube, PBS was added to the control tube. Thereafter, 5 μL of molecular antibodies (PE-CD45, PE-CD73, FITC-CD34, and FITC-CD90) was added, and the mixture was incubated for 30 min in the dark. The unbound antibodies were removed, and then flow cytometry was used to count the number of cells.

#### 2.3.4 Multilineage differentiation of BMSCs

Six-well plates were used to seed third-generation BMSCs at a cell density of 1.5 × 10^4^ per well. BMSC osteogenic induction medium, adipogenic induction medium, and chondrogenic induction medium (all from Cyagen, United States) were added for culturing once the cells had reached 80% confluence.

Cultures were terminated on day 21 and stained with alizarin red, oil red O, and alcian blue (Cyagen), respectively and subsequently observed under an inverted microscope.

#### 2.3.5 Mixed culture of NSCs and BMSCs on GelMA hydrogel surfaces

First, 96-well plates were filled with varied concentrations of GelMA hydrogels, which were then exposed to UV light for 12 s to cure them. Then, neurospheres and BMSCs were separated into individual cells using trypsin and mixed at a 1:1 ratio. The mixed cells were resuspended on GelMA hydrogel by seeding 2 × 10^3^ cells per well. The NSC full medium was added to the mixed cells after which they were cultivated in a humid incubator at 37°C with 5% CO_2_. The medium was replaced every other day. Cells were photographed at set time points.

#### 2.3.6 Cell migration assay

Co-cultured NSCs and BMSCs were seeded on the hydrogel scaffold. After 3 days of culture, the culture medium was removed and the cell scaffold was washed. Then, 4% paraformaldehyde (v/v) was used to fix the scaffolds for 30 min, and 0.5% Triton X-100 was used to infiltrate the cells for 20 min. The scaffolds were stained for 30 min in the dark with FITC-phalloidin (Invitrogen). Laser scanning confocal microscopy (LSCM, Olympus, Japan) was used to observe the depth of cell migration on the hydrogel.

#### 2.3.7 Photoencapsulation and 3D culture of co-cultured cells

Neurospheres and BMSCs were mixed in GelMA hydrogel at a cell concentration of 1 × 10^6^/mL. To create the cell-encapsulated hydrogel scaffold, the produced cell suspension was injected into a PMMA mold with a diameter and height of 5 mm and 2 mm, respectively. The UV light was then shone on the mould for 30 s. Cell scaffolds containing NSCs and BMSCs were transferred to NSC complete medium and cultured at 37° C in a 5% CO2 incubator.

#### 2.3.8 Cytocompatibility assessment

Cell scaffolds were cleaned and treated in the dark with the Calcein-AM/PI kit (Invitrogen) for 30 min after 3 days of *in vitro* growth with the cells encapsulated. After washing the scaffolds again with PBS, LSCM was used to visualise cell viability in the hydrogels.

On days 1, 4, and 7, mixed cell growth in the hydrogel was evaluated using the Cell Counting Kit-8 (CCK8, Invitrogen). Reagents containing 10% CCK-8 were added after the cell culture medium had been removed at different time points, incubated for 2 h, and then transferred to a 96-well culture plate. The absorbance was then determined at 450 nm using a microplate reader (BioTek, United States).

#### 2.3.9 Encapsulated cell immunofluorescence staining

Cell scaffolds were cleaned with PBS, fixed for 30 min with 4% paraformaldehyde, and then cleaned once more with PBS. The scaffolds were blocked with 5% BSA (Invitrogen) for a further 1 h after being infiltrated with 0.5% Triton X-100 for 30 min. Thereafter, Tuj-1 and GFAP primary antibodies were added, and they were incubated at 4°C overnight. The secondary antibody solution was added after the primary antibody had been removed by washing. Finally, DAPI was applied to stain for nuclei for 10 min, and the scaffolds were photographed under a fluorescence microscope.

#### 2.3.10 Western blotting analysis

To investigate the role of Notch signalling between NSCs and BMSCs, the cell scaffolds were divided into two groups. Based on the NSC complete medium, the experimental group was treated with 10 μM of the signalling pathway inhibitor DAPT (Sigma), whereas the control group received no treatment. The expression of Notch signalling pathway-related proteins in the two groups was detected after 2 days of culture.

The hydrogel scaffold of the encapsulated cells was placed in GelMA lysate (EFL, China), and after the cells were completely released from GelMA, the lysate was centrifuged at 4°C for 5 min. RIPA lysis buffer (Thermo Fisher Scientific, United States) was added after removing the supernatant, and after 30 min on ice, it was centrifuged for a further 20 min. The BCA protein assay kit (Thermo Fisher Scientific) was used to quantify the supernatant after it had been transferred to a centrifuge tube. Electrophoretic separation was performed on 30 μg of protein using SDS-PAGE (Thermo Fisher Scientific), and proteins were then transferred to PVDF membranes. After being blocked with 5% skim milk for 2 h at room temperature, the PVDF membrane was incubated with primary antibodies (Tuj-1, GFAP, Hes-1, ASCL-1, and β-actin) overnight at 4°C, washed three times with TBST (TBS with Tween-20, 1X), and then incubated for 2 h with secondary antibodies at room temperature. Finally, the immunoreactive bands were visualised using an enhanced chemiluminescence kit (Beyotime Biotechnology, Shanghai, China). The immunoreactive bands were analysed using ImageJ software.

### 2.4 *In vivo* testing

#### 2.4.1 Surgery procedures

All procedures followed the NIH Guide for the Care and Use of Laboratory Animals. The experiments were approved by the Institutional Animal Care and Use Committee of Bengbu Medical University. Ninety female SD rats weighing 200–240 g each were randomly assigned to one of six groups (n = 15 each), namely, the sham operation group (Sham group); spinal cord injury group (SCI group); GelMA transplantation group (GelMA group); GelMA encapsulated BMSC group (BMSCs group); GelMA encapsulated NSCs group (NSCs group); and GelMA encapsulated NSCs and BMSCs group (NSCs/BMSCs group).

As previously described (Liu et al., 2017), the SCI model was established. In brief, sodium pentobarbital (50 mg/kg) was used to anesthetise the rats. The surgical site was sterilised, the laminectomy was excised at the T8–T9 level, the paraspinal muscles were separated, and a 2 mm incision was made by hemisected right to the longitudinal axis of the spinal cord. The spinal cord tissue in the incision was removed, bleeding from the incision was stopped and the incision rinsed with normal saline, and the scaffold was implanted into the spinal cord defect area. The rats’ skin and muscle tissue were then layer by layer stitched. To avoid incision infection, chloramphenicol (50–75 mg/kg) was given to all rats for 7 days. After surgery, the bladder was manually emptied twice a day.

#### 2.4.2 Behavioural assessment

The recovery of lower limb motor function in the SCI rats was assessed using the Basso, Beattie and Bresnahan (BBB) motor assessment scale. Scores vary from 0 to 21, with 21 denoting normal motor function and 0 denoting total paralysis. The rats were placed in the open area to move freely, and the lower limb motor function of the rats was evaluated at 1–8 weeks post-surgery by two investigators unaware of the grouping.

#### 2.4.3 Tissue preparation and immunofluorescence

All animals were anesthetised with pentobarbital at week 8 post-surgery and perfused transcardially with precooled PBS and 4% paraformaldehyde. A 2-cm spinal cord tissue centred at the site of injury was isolated and immersed overnight in 4% paraformaldehyde followed by dehydration in 30% sucrose buffer. The tissues were embedded with OCT (Sakura FineTec Inc., Japan), and six groups of spinal cord tissues were cut longitudinally on a freezing microtome, each with a thickness of approximately 8 μm.

The sections were blocked in BSA blocking solution for 1 h, following which primary antibodies (Tuj-1, GFAP, CS-56, and Laminin) were added after the blocking solution was removed via aspiration, and incubated overnight at 4°C. After the primary antibody incubation was finished, the sections were washed with PBS before being treated with the secondary antibody for 2 h in the dark. Finally, sections were sealed with DAPI containing anti-fluorescence quencher (Maravai LifeSciences, United States). An inverted fluorescence microscope was used to capture the images, and ImageJ was used to tally the mean fluorescence intensity.

#### 2.4.4 Histological analysis

Spinal cord sections were prepared according to the procedure of tissue immunofluorescence staining. The sectioned tissues were rinsed with PBS, stained with haematoxylin and eosin (H&E), and photographed using an inverted microscope. The structure of the spinal cord was observed, and the percentage of the total volume of the injured area was measured. By dividing the entire volume of the wounded area by the corresponding spinal cord volume, the percentage of the total volume of the injured area was computed.

### 2.5 Statistical analysis

Data are expressed as the mean ± standard deviation. One-way analysis of variance (ANOVA) was performed to assess the statistical significance between groups, whereas GraphPad Prism software (San Diego, California, United States) was used for statistical analysis. Statistical significance was considered at P < 0.05.

## 3 Results

### 3.1 GelMA hydrogel characterisation

The microstructure within the hydrogel showed a porous network for all three types of GelMA hydrogels (5, 10, and 15% w/v) as observed through SEM (Figures 2B–D). As the concentration of the hydrogel increased, so did the thickness of the pore wall gradually. The average thicknesses of the pore walls of the 5%, 10%, and 15% (w/v) GelMA hydrogels were 2.0 ± 0.58 μm, 6.0 ± 0.57 μm, and 10.3 ± 1.45 μm, respectively (Figure 2E). The compression modulus was positively correlated with the concentration of the hydrogel; the 5% GelMA hydrogel had the lowest compression modulus (4.4 ± 0.41 kPa), whereas the 15% GelMA hydrogel had the highest compression modulus (45.4 ± 1.79 kPa) (Figure 2F).

*In vitro* degradation experiments revealed that the degradation of the three hydrogels decreased as GelMA concentration increased (Figure 2G). After 2.5 h, the 5% GelMA was entirely degraded; the 10% and 15% GelMA, while experiencing substantial volume reduction after 3 h of cultivation, retained 23.4% and 32.2% of their weight/volume, respectively. To assess the swelling rate of the three GelMA hydrogels within 48 h, the ratio of wet weight to dry weight for the three hydrogel groups was determined at different time points during swelling (Figure 2H). The swelling capacity for the 5% and 10% hydrogels peaked around 24 h then stabilised without further swelling. The 15% hydrogel, however, required a longer period to reach equilibrium, approximately 36 h.

The hydrogel wettability was assessed using water contact angles. As GelMA hydrogel concentration increased, so did the water contact angle, with the 5% GelMA hydrogel exhibiting the highest wettability (Figure 2I). To compare the water content across different concentrations of hydrogels, it was necessary to study the water retention capacity of the scaffolds. Remarkably, the water absorption of the 5% GelMA hydrogel was >13 times its own weight, considerably exceeding the 6 times weight absorption of the 15% GelMA. Throughout the experiment, the 5% GelMA hydrogel showed a consistently higher water absorption rate than the other two groups (Figure 2J).

### 3.2 *In vitro* studies

#### 3.2.1 Evaluation of isolated NSCs

Cultured NSCs proliferated from single cells to form small clusters before the formation of larger suspended neurospheres (Supplementary Figure S1A1–A3). Immunofluorescence staining of the neurospheres showed positive staining for the NSC marker Nestin (Supplementary Figure S1B).

#### 3.2.2 Evaluation of isolated BMSCs

BMSCs were extracted from the bone marrow that was extracted from the tibia of GFP-expressing rats. Under the inverted microscope, we observed the cultured BMSCs adhering to the culture dish, exhibiting a long, spindle-shaped morphology and forming clusters (Supplementary Figure S2A1–A3). Upon *in vitro* induction, these BMSCs showed positive staining for osteogenic, adipogenic, and chondrogenic differentiation (Supplementary Figure S2B1–B3). Flow cytometry showed low expression of CD-34 (66.5%) and CD-45 (58.8%), and high expression of CD-73 (99.4%) and CD-90 (99.4%) (Supplementary Figure S2C).

#### 3.2.3 BMSCs promote the differentiation of neurosphere cells *in vitro*

**FIGURE 3:**
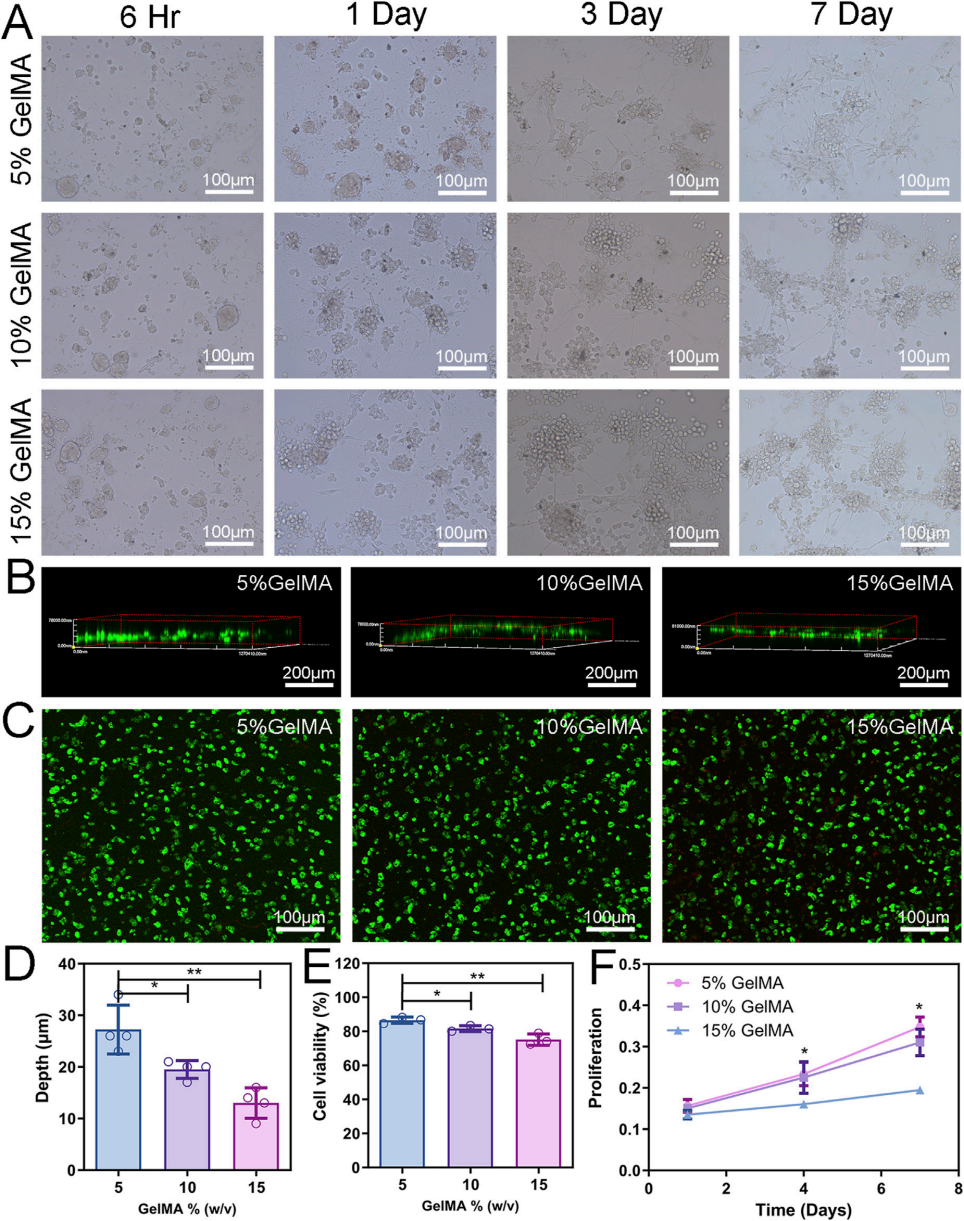
2D Cell co-culture and 3D cytocompatibility. **(A)** Cell morphology of NSCs and BMSCs co-cultured on the hydrogel surface with different concentrations. **(B)** LSCM was used to observe the depth of cells entering the scaffold. **(C)** The activity of co-cultured cells in the hydrogel was detected by Calcein-AM/PI staining. Green fluorescence (AM) indicates viable cells and red fluorescence (PI) indicates dead cells. **(D)** Analysis of cell migration depth. **(E)** Assessment of cell viability. **(F)** Proliferation of cells encapsulated in different concentrations of hydrogels. (n = 3; *p < 0.05, **p < 0.01).

Upon co-culturing NSCs and BMSCs, cells from the neurospheres adhered to the bottom of the GelMA hydrogel (Figure 3A). Over time, neurites of the neurosphere cells extended as early as 6 h after co-culturing, indicating rapid cell growth and interconnection. After 7 days of culture, cells within the hydrogel of the three groups formed different network structures.

#### 3.2.4 Infiltration, viability, and cell proliferation inside the hydrogels

NSCs and BMSCs were injected onto the hydrogel scaffold’s surface to assess the capacity of the cells to migrate inside it. After 3 days of culture, cells were stained with phalloidin, and the 3D reconstruction image of the LSCM showed that the depth of cell infiltration on the 5% GelMA scaffold was 27.3 ± 2.36 µm, significantly higher than that of 10% GelMA (19.5 ± 0.87 µm) and 15% GelMA (13.0 ± 1.47 µm) (Figures 3B, D).

The cytocompatibility of cells with the hydrogel was assessed through live/dead cell staining and CCK-8 assay. After 3 days of *in vitro* culture of the 3D hydrogel scaffold, fluorescent imaging showed that the cell viability of cells grown in the 5% GelMA was 86.5%, significantly higher than that of the 10% (81.6%) and 15% (75.1%) GelMA hydrogels (Figures 3C, E), and gradually decreased with the increase in hydrogel concentration. Supplementary Figure S3 details images of live cells, dead cells, and their staining. To assess the proliferation of co-cultured cells, cell proliferation in the hydrogel scaffold at 1, 4, and 7 days was determined using the CCK-8 reagent. Cell proliferation within the hydrogel significantly decreased with the increase in hydrogel concentration. Cell proliferation in the 5% GelMA hydrogel scaffold was higher at all three time points compared with the other two groups (Figure 3F). These results indicate that hydrogel scaffolds with a low modulus are more effective in facilitating cell migration and compatibility, and can provide superior microenvironment support for cell survival.

#### 3.2.5 Effect of hydrogel modulus on cell differentiation and axon growth in co-culture

**FIGURE 4:**
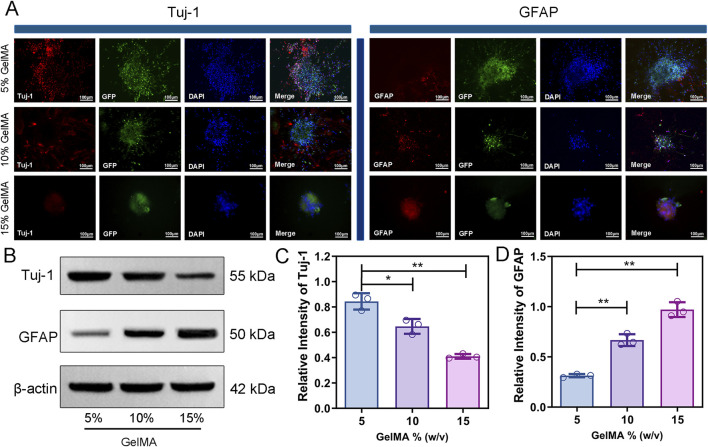
GelMA hydrogel with low modulus promotes neuronal differentiation and inhibits GFAP expression. **(A)** Immunofluorescence staining images of Tuj-1 and GFAP of co-cultured cells encapsulated with three different concentrations of hydrogels for 7 days. **(B–D)** Protein expression and quantitative analysis of Tuj-1 and GFAP on hydrogel of each group. (n = 3; *p < 0.05, **p < 0.01).

The differentiation of co-cultured cells in hydrogel was monitored by immunofluorescence labelling. In the cells cultured within the 5% GelMA hydrogel, the expression level of Tuj-1, a marker for neuronal differentiation, was significantly higher than that in the other two groups (Figure 4A). In addition, extensive neurite growth was observed in the 5% GelMA hydrogel, with the longest cell extensions reaching up to 249.5 μm, whereas the neurospheres in the 15% hydrogel remained spherical (Supplementary Figure S4). Immunofluorescence analysis showed that GFAP expression, a marker for astrocyte differentiation, was highest in the 15% GelMA hydrogel and lowest in the 5% GelMA hydrogel (Figure 4A).

The findings of the Western blot analysis of Tuj-1 and GFAP expression in various hydrogel concentrations were in agreement with the immunofluorescence images (Figure 4B). The expression of Tuj-1 in the 5% GelMA hydrogel was significantly higher than that in the 10% and 15% GelMA hydrogel groups, with higher scaffold concentrations corresponding to lower Tuj-1 expression (Figure 4C). The GFAP expression showed an opposite trend to that of Tuj-1, where the higher the hydrogel concentration, the higher the GFAP expression in the cell-laden scaffold (Figure 4D). These results indicate that co-cultured cells in a low-modulus hydrogel can enhance neuronal differentiation and inhibit the proliferation of astrocytes. Since the 5% GelMA hydrogel showed excellent material properties, high cell compatibility, and great ability to promote stem cell differentiation, we selected a 5% hydrogel ratio for the subsequent work.

#### 3.2.6 Notch signaling mediates the differentiation of NSCs

The pathway through which BMSCs influence NSC differentiation was analysed using Western blot. Two days after co-culturing the two groups of cells, several strip-like structures and neuron-like protrusions could be observed in the control group under an inverted microscope, whereas the number of strip-like structures decreased after adding the DAPT inhibitor. Western blot analysis of the two groups of cells showed that Hes-1 expression levels in the control group were greater than those in the DAPT inhibitor group, whereas ASCL-1 expression was much lower in the control group than it was in the DAPT inhibitor group (Supplementary Figure S5A, B). The findings show that the Notch signaling pathway is essential for NSC proliferation and differentiation when BMSCs are involved.

### 3.3 *In vivo* testing

#### 3.3.1 Reduce lesion volume and improve motor function recovery

**FIGURE 5:**
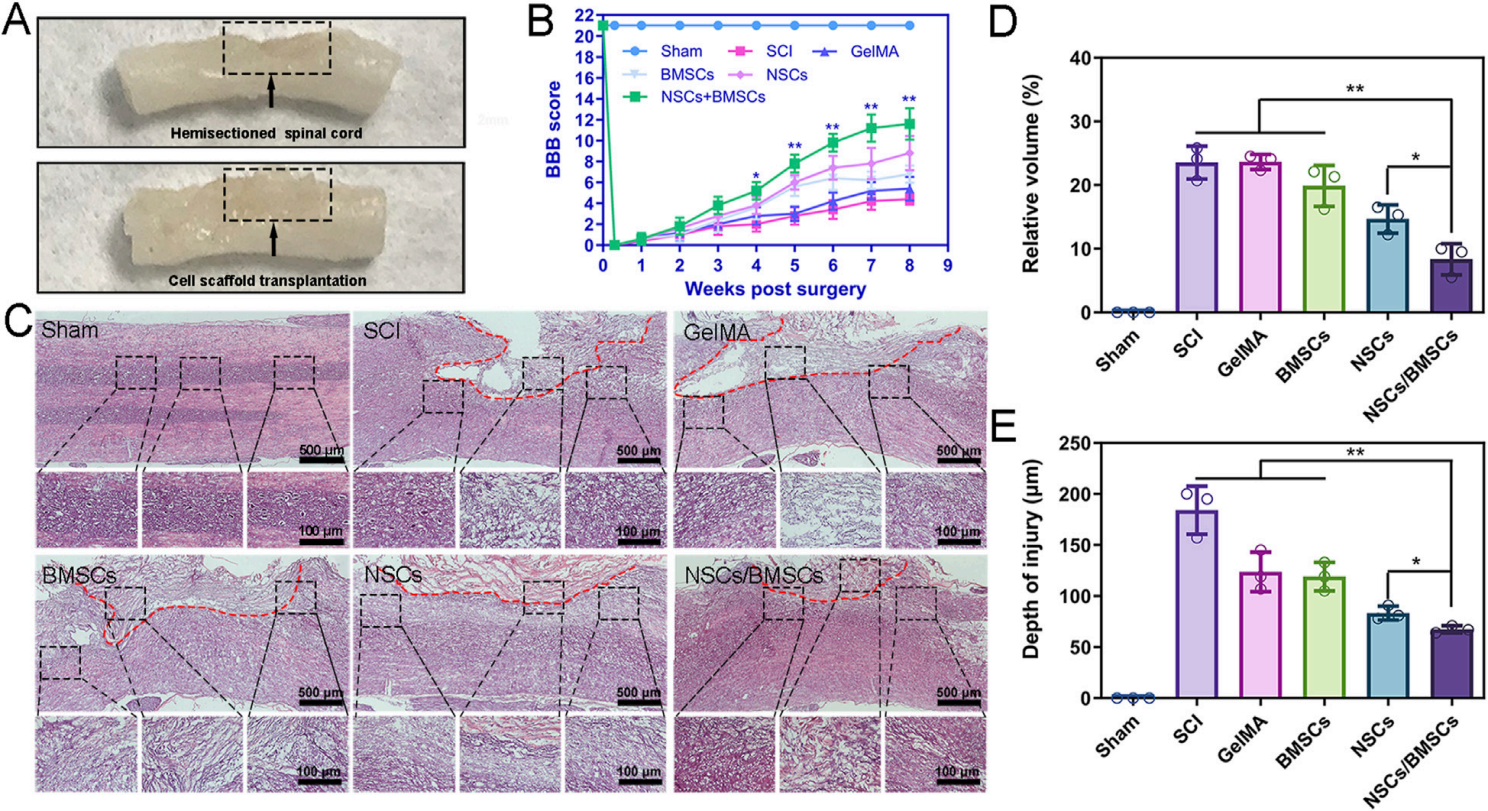
Co-cultured cell scaffolds improve the pathological and motor function of SCI rats. **(A)** Representative images after treatment of SCI. **(B)** BBB scores of hind limbs of rats in each group. **(C)** HE staining of spinal cord longitudinal sections at low (top) and high (bottom) magnification at 8 weeks after surgery. **(D–E)** Percentage of spinal cord lesion cavity volume and lesion depth in each group. (Biological repeats, n = 3; *p < 0.05, **p < 0.01).

SCI can cause lower limb motor dysfunction and the regenerative effects of scaffolds on SCI can be evaluated through the restoration of lower limb function (Figures 5A, B). After a hemisection of the right side of the spinal cord, the right hind limb of the rat immediately became completely paralysed (Figure 5B). In the first 3 weeks following SCI, slight recovery of motor function was observed in all five groups of SCI rats, but no significant difference in scores was observed. Starting from week 4 post-surgery, the scores of the NSCs/BMSCs group significantly differed from the other groups. By week 8, the NSCs/BMSCs group had a score of 11 points, which was much more than the combined scores of the other 4 groups. The group of co-cultured cells exhibited a more favourable effect on the repair of SCI than either NSCs or BMSCs alone. These results suggest that the GelMA hydrogel loaded with NSCs/BMSCs improved the functional recovery following SCI.

At week 8 post-surgery, we observed the structure of spinal cord tissues and lesion volume using H&E staining and found large areas of tissue interruption and vacuolisation at the injury location of the SCI and GelMA groups, with considerable disruption of the tissue structure (Figure 5C). After cell scaffold implantation, all hydrogel scaffolds degraded, and both NSCs and BMSCs groups, as well as the co-cultured NSCs/BMSCs group, displayed reduced cavity formation. The volume of lesion cavity of each group in the H&E sections was measured as follows: 23.6% in the SCI group, 23.5% in the GelMA group, 19.8% in the BMSCs group, 14.7% in the NSCs group, and 8.3% in the NSCs/BMSCs group (Figure 5D).

The NSCs/BMSCs group not only showed reduced cavity formation but also further limited the spread of spinal cord lesions. The depth of the lesions was 67.3 μm for the NSCs/BMSCs group, lower than the BMSCs (119.0 μm) and NSCs (83.3 μm) groups (Figure 5E). These results confirm that the NSCs/BMSCs-GelMA hydrogel can reduce the cavity area, limit lesions at the injury site, and promote cell infiltration and tissue formation.

#### 3.3.2 Promotion of NSCs differentiation

**FIGURE 6:**
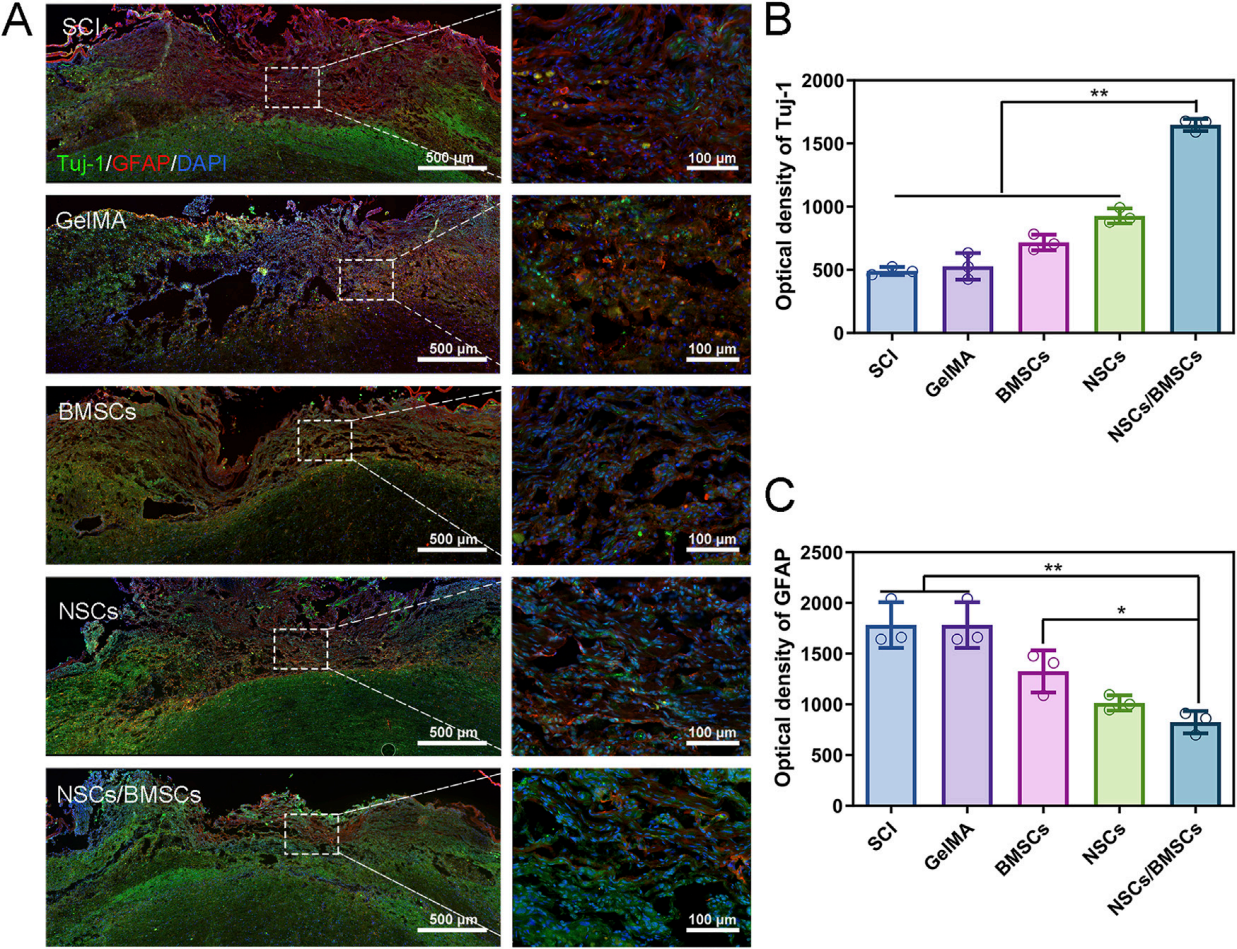
Enhancement of nerve regeneration by co-cultured cell scaffolds. **(A)** Immunofluorescence staining of Tuj-1 positive axons (green) and GFAP astrocytes (red) at the injury site of rats in each group 8 weeks after injury. **(B–C)** Quantitative analysis of fluorescence intensity of Tuj-1 and GFAP (n = 3; *p < 0.05, **p < 0.01).

Immunofluorescent staining was used to detect the expression of the neuronal marker Tuj-1 and the astrocyte marker GFAP in spinal cord tissue (Figure 6). At week 8 post-surgery, the distribution of Tuj-1 and GFAP was observed in all groups, but the greatest quantity of Tuj-1-labelled neurons at the injury site was observed in rats from the NSCs/BMSCs group (Figures 6A, B). The NSCs and BMSCs groups had considerably more Tuj-1 positive neurons at the damage site than the pure GelMA group. The expression of Tuj-1 in the pure SCI group was almost indistinguishable. The expression of GFAP was opposite to that of Tuj-1 (Figure 6C). Both the SCI and GelMA groups showed a significant amount of GFAP-positive astrocytes. However, the proliferation of reactive astrocytes significantly decreased after co-culturing with GelMA hydrogel, with the most pronounced effect seen in the NSCs/BMSCs group. Therefore, GelMA hydrogel loaded with NSCs/BMSCs can encourage neuronal development and inhibit astrocyte proliferation *in vivo*.

#### 3.3.3 Suppression of scar formation

**FIGURE 7:**
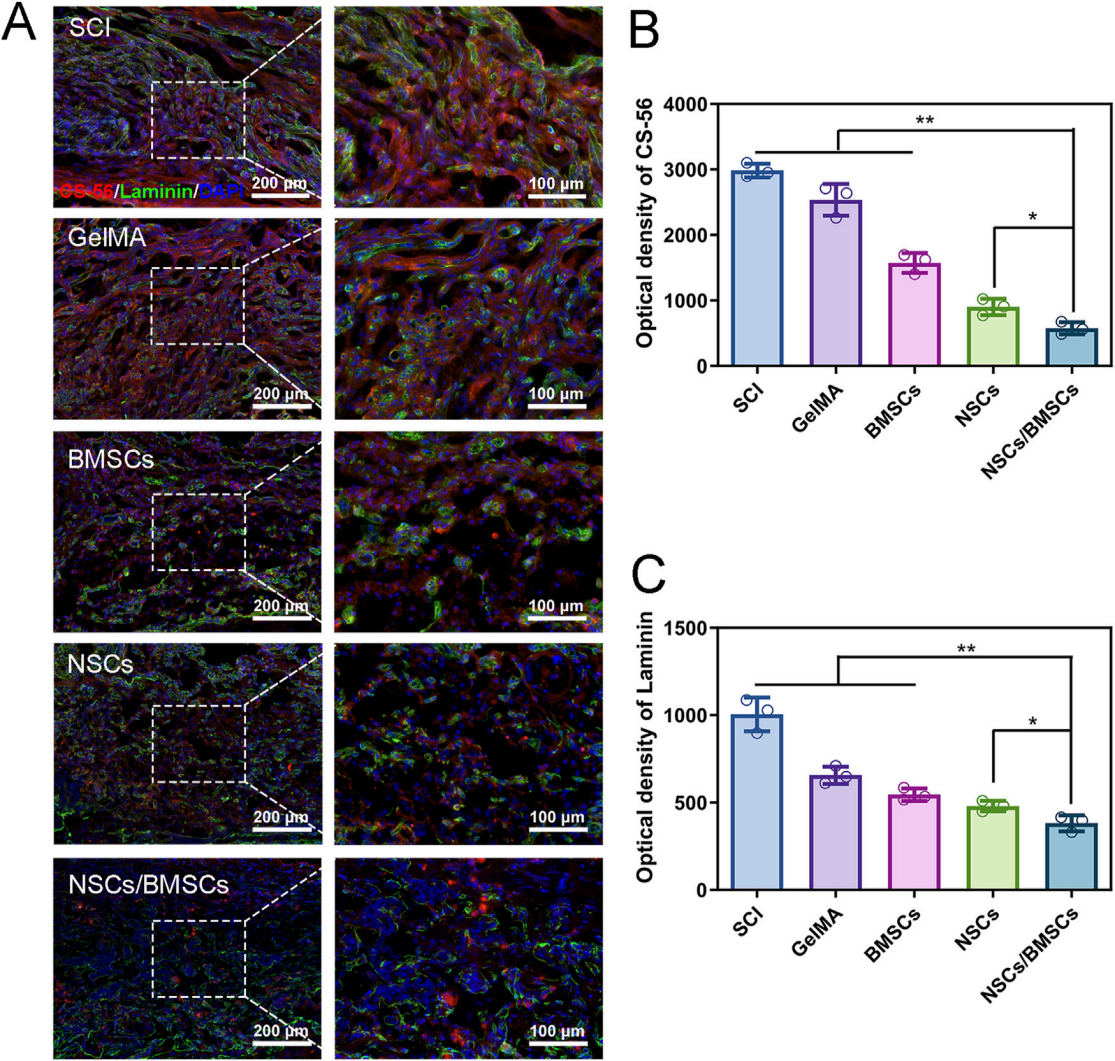
Inhibition of scar tissue formation by co-cultured cell scaffolds. **(A)** Immunofluorescence staining of CS-56 labeled glial scar (green) and Laminin labeled fibrous macula (red) at the injured site of each group. **(B–C)** Fluorescence quantitative analysis of CS-56 and Laminin. (n = 3; *p < 0.05, **p < 0.01).

The scar is a physical barrier to neural regeneration and axonal growth, which primarily includes glial scars and fibrotic scars. Immunofluorescent staining was used to assess the deposition of CS-56-labelled glial and Laminin-labelled fibrotic scars following SCI (Figure 7). Glial scars are formed by the astrocytes-secreted chondroitin sulphate proteoglycans. The glial scars labelled with CS-56 were less in the NSCs/BMSCs group compared with the other treatment groups (Figures 7A, B). Single transplantation of NSCs or BMSCs also had an inhibitory effect on glial scars, but a large number of glial scars were present in the SCI and GelMA groups. Fibroblasts secrete extracellular matrix molecules which can support the formation of a fibrotic scar. The results of Laminin staining were consistent with the CS-56 results, indicating that the fibrotic scars formed in the NSCs/BMSCs group were significantly less than those in the SCI and GelMA groups (Figure 7C). The results show that NSCs/BMSCs-laden GelMA hydrogel scaffolds can effectively inhibit the formation of scar tissue.

## 4 Discussion

SCI can lead to secondary structural complications, inflammation response, apoptosis, and glial/fibrotic scar formation at the site of injury. Due to the limited regenerative ability of the central nervous system (CNS), strategies for SCI repair remain limited (Liu et al., 2021; Wei et al., 2021). The presence of endogenous neural stem/progenitor cells (eNSPCs) within the spinal cord offers a promising and non-invasive therapeutic avenue for addressing spinal cord injuries. Targeted interventions have the potential to induce eNSPCs to differentiate into neurons, construct neural networks, and form oligodendrocytes (Li et al., 2023). Nevertheless, the post-injury microenvironment in the spinal cord is suboptimal, which diminishes the differentiation capacity of endogenous eNSPCs. Furthermore, the population of eNSPCs is markedly reduced in the spinal cords of adult rats, thereby exacerbating the challenges associated with spinal cord injury repair (Stenudd et al., 2015). Various stem cell sources have been employed to heal SCI, with stem cell-based transplantation being the major focus of recent advancements in SCI treatment (Assinck et al., 2017). Due to the easy availability of NSCs and their homogeneity with *in situ* tissues, they are considered one of the best options for treating neurological diseases. The basic principle of using NSCs in SCI treatment is to replace the lost tissue (mainly neurons and oligodendrocytes) through these cells and provide nutritional support for surviving neural tissues (Vismara et al., 2017). However, when NSCs are transplanted into the healthy or damaged spinal cord of adult rats, they either remain undifferentiated or differentiate along the glial lineage (Xue et al., 2021). The role of MSCs in promoting the differentiation of NSCs has been previously demonstrated. By co-culturing BMSCs and NSCs, the two types of stem cells can interact. BMSCs can support the transplantation of NSCs through neuroprotection, immune regulation, and pro-angiogenic activities (Han and Hsu, 2017; Li et al., 2021; Kaminska et al., 2022). BMSCs possess the ability to induce neuronal differentiation of NSCs while concurrently enhancing the proliferation and survival of NSCs, as well as reducing the incidence of tumours caused by stem cell transplantation (Zhou et al., 2010; Yang et al., 2021).

Several limitations must be addressed prior to the clinical application of cell therapy, namely, the short survival time of cells in the injured area, low cell activity, and uncontrolled differentiation. The long-term progress of tissue engineering showed that the rational design of biomaterials can address these challenges and improve the efficacy of stem cell therapy. A suitable biomaterial has several key features: it responds to the physicochemical properties of the microenvironment, boasts high biocompatibility, and exhibits low immunogenicity (Cao et al., 2021; Walsh et al., 2022). Many biocompatible and bioactive hydrogel scaffolds, such as collagen, chitosan, or hyaluronic acid hydrogels, have been shown to enhance the survival and even proliferation of transplanted stem cells (Liu et al., 2022; Shen et al., 2022). Although GelMA based on gelatin is widely used in the field of tissue engineering (Monteiro et al., 2018; Bahcecioglu et al., 2019), its usage in SCI predominantly involves the delivery of a single type of therapeutic cell (Fan et al., 2018) or functioning as a supporting extracellular matrix to promote axonal regeneration (Koffler et al., 2019; Ma et al., 2020). Data on the impact of GelMA on co-cultured cells are limited. In this study, we developed a 3D hydrogel scaffold system encapsulating NSCs and BMSCs based on the photosensitive GelMA hydrogel scaffold. We demonstrated that the low-modulus GelMA hydrogel sustained the survival of co-cultured cells *in vitro* while promoting cell proliferation, migration, and neuronal differentiation. Furthermore, the motor function scores of the co-cultured group of cell scaffold were significantly higher than those of other treatment groups. This cell scaffold reduced the cavity area and the inflammatory response in the SCI region, increased neuronal expression, and inhibited scar tissue formation. These findings confirmed that co-cultured cells, when loaded on low-modulus scaffold materials, can stimulate neural regeneration and the formation of subsequent functional neural networks.

CNS neurons and glial cells are extremely sensitive to mechanical factors and prefer “soft” substrates that mimic the mechanical characteristics of the *in situ* tissue microenvironment (Moshayedi et al., 2014; Koser et al., 2016). Hydrogel mechanical characteristics significantly impact cell survival, neural development, and axon creation (Pek et al., 2010; Tseng et al., 2015). At the Young’s modulus of the substrate ranging between 0.1 and 1 kPa, NSCs tend to differentiate into neurons, whereas at a range of 1–10 kPa, they are more likely to differentiate into glial cells (Engler et al., 2006; Nagai et al., 2012). For example, Fan et al. demonstrated that primary mouse-induced multipotent stem cells grown on a soft substrate (0.68 kPa), compared with a harder substrate (2.03 kPa), had significant therapeutic effects in promoting axonal regeneration while inhibiting glial scar formation (Fan et al., 2018). The Young’s modulus of the 5% GelMA hydrogel used in this study was 4.4 ± 0.41 kPa, within the range of CNS biocompatibility. Moreover, the porous microstructure of the hydrogel and the relatively low pore wall thickness facilitate the migration of nutrients within the network, a key characteristic for the delivery of therapeutic substances. The average pore wall thickness of 5% GelMA was 2.0 ± 0.58 µm, considerably lower than the other two groups of hydrogels, which favours the infiltration of neural cells and neuronal fibres, as well as the migration of larger therapeutic biomolecules (Sun et al., 2019b; Walsh et al., 2022).

Biodegradability represents a notable advantage in the using hydrogels in the field of tissue engineering. Hydrogels should not only support cell proliferation and differentiation but should also balance hydrogel degradation with tissue regeneration (Chen et al., 2019). Degradation experiments showed that the lower the GelMA concentration, the greater the scaffold weight loss. This might be explained by low-concentration GelMA hydrogels, due to their lower methacrylamide ratio, have scaffolds more susceptible to breakage after enzyme action following photopolymerisation, which provides space for the growth of internal tissues. The swelling characteristics of hydrogels in tissue engineering applications reflect the water absorption capacity of the hydrogel and can further predict its degradation rate (Browning et al., 2014). The size of the polymer network’s pores and how the polymer and solution interact determine how much hydrogels expand. Studies on all three hydrogel concentrations showed that reducing polymer concentration increased the mass swelling rate of the hydrogel, and the time required for low-concentration hydrogels to reach equilibrium was shorter. An increase in cross-linking density of high-concentration hydrogels not only limits the penetration of solution inside the hydrogel but also slows down hydrogel degradation (Lam et al., 2014). In addition, 5% GelMA hydrogel has higher water content and wettability, which is conducive to the transport of nutrients and metabolites, as well as beneficial for cell survival (Seliktar, 2012). These material parameters indicate that low-modulus hydrogels have good physicochemical properties, which are conducive to the maintenance of cell viability and proliferation of encapsulated cells.

We believe that the high porosity and low wall thickness of low-modulus hydrogels can reduce cell migration resistance. More significantly, cells in soft hydrogel scaffolds have a propensity to recognise, sense, and attract a greater number of cell binding sites (Nichol et al., 2010; Shi et al., 2022). The 3D image reconstruction of cell scaffolds through LSCM showed that the migration depth of NSCs/BMSCs on 5% GelMA scaffolds was significantly higher than on 10% and 15% GelMA gel scaffolds. Viability assay of NSCs and BMSCs co-cultured for 3 days was performed to assess the 3D cell viability. Almost no dead cells were found in any of the three concentrations of GelMA scaffolds used, and 5% GelMA significantly improved the activity of cells in the 3D hydrogel, showing superior performance with a higher proportion of healthy cells (green). By detecting cell proliferation in the three concentrations of hydrogels, we found that cell proliferation in 5% GelMA was significantly higher at different time points than in the other two hydrogels. Therefore, we believe that the co-cultured cells encapsulated in low-modulus GelMA hydrogel have superior bioactivity.

Through an examination of the cell behaviour of NSCs/BMSCs in the scaffold, we assessed the function of the cell scaffold in promoting neural tissue regeneration. Immunofluorescence staining of the cells in the scaffold showed that the cell morphology in the 5% hydrogel was spread out, and the free diffusion of cells provided a permanent 3D microenvironment for molecular exchange between cells. Almost no pseudopodia were generated in the cells in the 15% GelMA hydrogel. In terms of cell differentiation, the protein expression of Tuj-1 and GFAP in the three groups of hydrogels was tested. After encapsulation of cells for 7 days, the content of Tuj-1 in the 5% GelMA group was 1.3 and 2.1 times higher than in the 10% and 15% GelMA groups, respectively. GFAP in the low-modulus GelMA showed a trend opposite to that of Tuj-1, with GFAP being 0.5 and 0.3 times that of the other two groups, respectively. Compared with harder hydrogel scaffolds, soft hydrogel matrix is more conducive to the neural differentiation of NSCs (Wang et al., 2010). These results suggest that co-culturing cells in GelMA hydrogel, mechanical properties of which are similar to natural CNS tissue, provides a more suitable microenvironment for cell survival and neuron differentiation. The exceptional qualities of 5% GelMA make it a potential scaffold material for the regeneration of neural tissue.

H&E staining was carried out at week 8 after SCI to assess the implant’s ability to bridge tissue. All therapy groups demonstrated various degrees of neural tissue reconnection in comparison to the lesion cavity of the SCI group. Although GelMA hydrogel scaffold has tissue support functions, the lack of stem cells means that it cannot improve the integration of spinal cord tissue (Fan et al., 2018). GelMA loaded with purely NSCs or BMSCs significantly improved the tissue bridging effect of the scaffold, but most cells at the injury site exhibited the morphology of glial cells rather than neurons, and severe necrotic cells spread to surrounding tissues, indicating that the tissue reconnection effect of these two groups was poor. The scaffold loaded with NSCs/BMSCs effectively inhibited the diffusion of the lesion cavity and promoted nerve fibre regeneration and tissue integration, and the morphology and arrangement of regenerated neural tissue were most similar to functionally regenerated tissue.

NSCs/BMSCs loaded within the GelMA hydrogel promote neural regeneration and functional recovery. The transplantation of exogenous stem cells promotes axon regeneration and functional recovery, mainly due to the hydrogel providing a new regeneration site for nerve fibres (Li et al., 2016). In this study, we observed significant differences by quantitatively analysing the regeneration of Tuj-1 positive axons in different areas of each treatment group. Although extensive Tuj-1 positive axonal growth was visible in the injury site of cell transplantation groups, more new nerve fibres were found to extend into the injury site in the NSCs/BMSCs group. Compared with the NSCs/BMSCs group, hydrogels loaded with pure NSCs or BMSCs typically showed a smaller proportion of positive neurons *in vivo* and failed to regenerate neural tissue function effectively. However, their effectiveness suggests that the implantation of the scaffold provides a preliminary basis for long-term recovery of the lesion. The introduction of co-cultured cells promoted the effect of the scaffold, indicating that synergistic action is necessary for effective regeneration. In addition, the hydrogel scaffold loaded with cells led to a considerable reduction in GFAP, a marker for astrocytes at the injury site, whereas the performance of the co-cultured cell group was superior.

Scar tissue following SCI primarily consists of glial and fibrous scars; scar tissue formation can be observed through CS-56 labelled glial scars and Laminin-labelled fibrotic scars (Chen et al., 2018). Immunofluorescence results showed that the SCI group and GelMA exhibited clear glial scar and fibrous scar barriers, and the NSCs/BMSCs scaffold had a good inhibitory effect on the overactivity and aggregation of astrocytes and fibroblasts. In the growth inhibitory microenvironment of SCI, chondroitin sulfate proteoglycans (CSPGs) formed by GFAP form a repair barrier during SCI repair and inhibit the treatment of SCI. Co-transplanted cultures reduced GFAP differentiation and consequently CSPGs formation, so that CS-56 marker was significantly reduced in NSCs/BMSCs scaffolds. Excessive deposition of extracellular matrix is the main cause of fibrotic scar formation. It inhibits axonal growth outside the lesion site, and co-transplanted cultures provide a supportive microenvironment that reduces fibrotic scar formation. The aforementioned findings demonstrate that combined transplantation can significantly enhance the microenvironment of the injured spinal cord, inhibit the scar tissue formation, and promote neurogenesis, thereby promoting neural function recovery in SCI rats.

This study is subject to several limitations. Firstly, additional verification is required to ascertain whether BMSCs within the co-transplanted stent can persist or are eliminated following SCI treatment. Secondly, the absence of observed staining for mature neurons post-treatment necessitates further investigation and validation in future research endeavors.

## 5 Conclusion

In summary, our research suggests that encapsulating NSCs and BMSCs within a low-modulus GelMA hydrogel scaffold, and subsequently transplanting this co-cultured cell scaffold into the site of spinal cord injury, promotes repair of the injury. *In vitro* studies indicate that structurally optimised GelMA hydrogel can simulate the structure and mechanical properties of spinal cord tissue. The low-modulus GelMA hydrogel has favourable mechanical characteristics, degradation capacity, and biocompatibility, providing a conducive microenvironment for the survival of co-cultured cells, which in turn promotes the differentiation of NSCs into neurons and inhibits the proliferation of astrocytes. Following the transplantation of the cell scaffold into the injury site of rats with spinal cord injuries, significant improvements in the hind limb motor function were observed in the NSCs/BMSCs group. This manifested as a reduction in the cavity area, inhibition of scar tissue formation, and promotion of neural tissue regeneration. Based on our findings, it can be inferred that a promising therapeutic strategy for spinal cord injury involves the combined application of low-modulus hydrogel and co-cultured cells.

## Data Availability

The original contributions presented in the study are included in the article/Supplementary Material, further inquiries can be directed to the corresponding authors.
