# Supplementary material for: Co-culturing neural and bone mesenchymal stem cells in photosensitive hydrogel enhances spinal cord injury repair
Source: Front Bioeng Biotechnol. 2024 Dec 16;12:1431420. doi: 10.3389/fbioe.2024.1431420 (PMC11684404; doi:10.3389/fbioe.2024.1431420)
Supplement: Supplementary file 1 [file DataSheet1.doc]

**Co-culturing neural and bone mesenchymal stem cells in photosensitive hydrogel enhances spinal cord injury repair**

Jianzhong Bai^a,b,1,*^, Guoping Liu^a,c,1^, Yang Gao^b^, Xishan Zhang^b^, Guoqi Niu^d^, Hongtao Zhang^a,*^

^a^ Department of Orthopedics, the First Affiliated Hospital of Soochow University, Soochow University, Suzhou, Jiangsu, 215000, China.

^b^ Department of Orthopedics, The Second Affiliated Hospital of Shandong First Medical University, Tai'an, 271000, China.

^c^ Department of Spine Surgery, The Second Affiliated Hospital, Hengyang Medical

School, University of South China, Hengyang, Hunan, 421000, China.

^d^ Department of Orthopedics, The Second Affiliated Hospital of Bengbu Medical University, Bengbu, Anhui, 233000, China.

^*^ Corresponding author.

Jianzhong Bai (jianzhongb@163.com), Hongtao Zhang ([htzhangsz@126.com](mailto:htzhangsz@126.com)).

^1^ These authors contributed equally to this work.


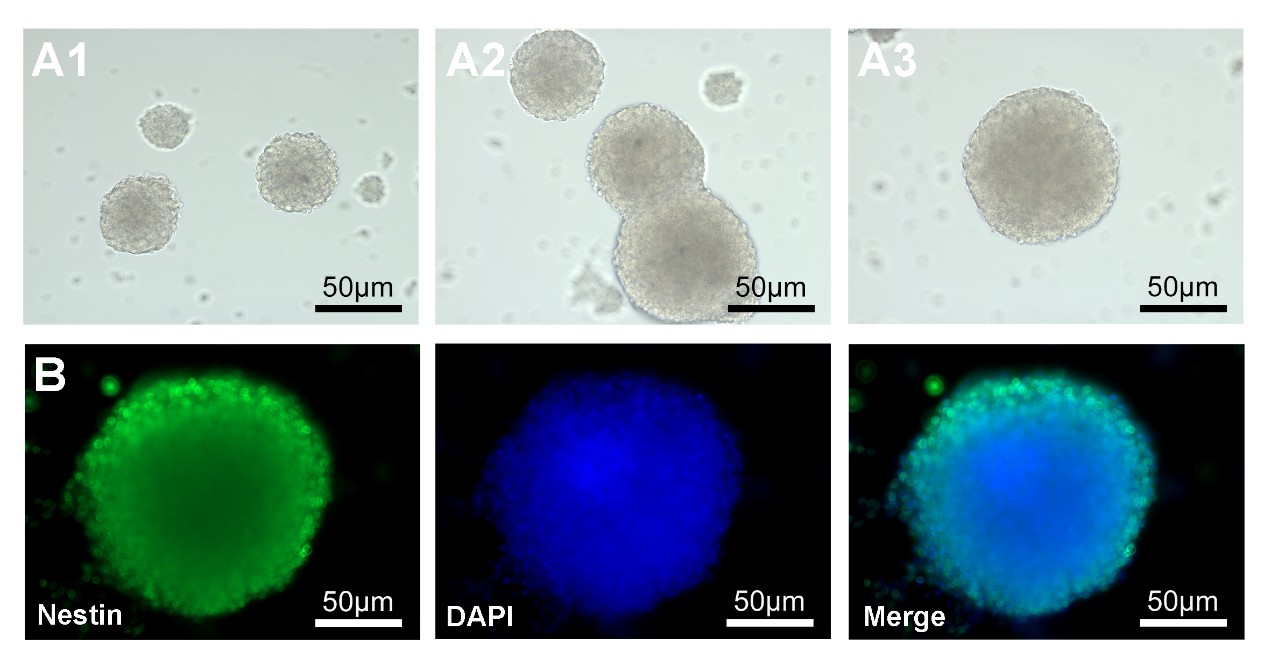


**Fig. S1.** Culture and identification of NSCs. (A1-A3) Morphological observation of NSCs. (B) Nestin staining was positive for the specific marker of NSCs.


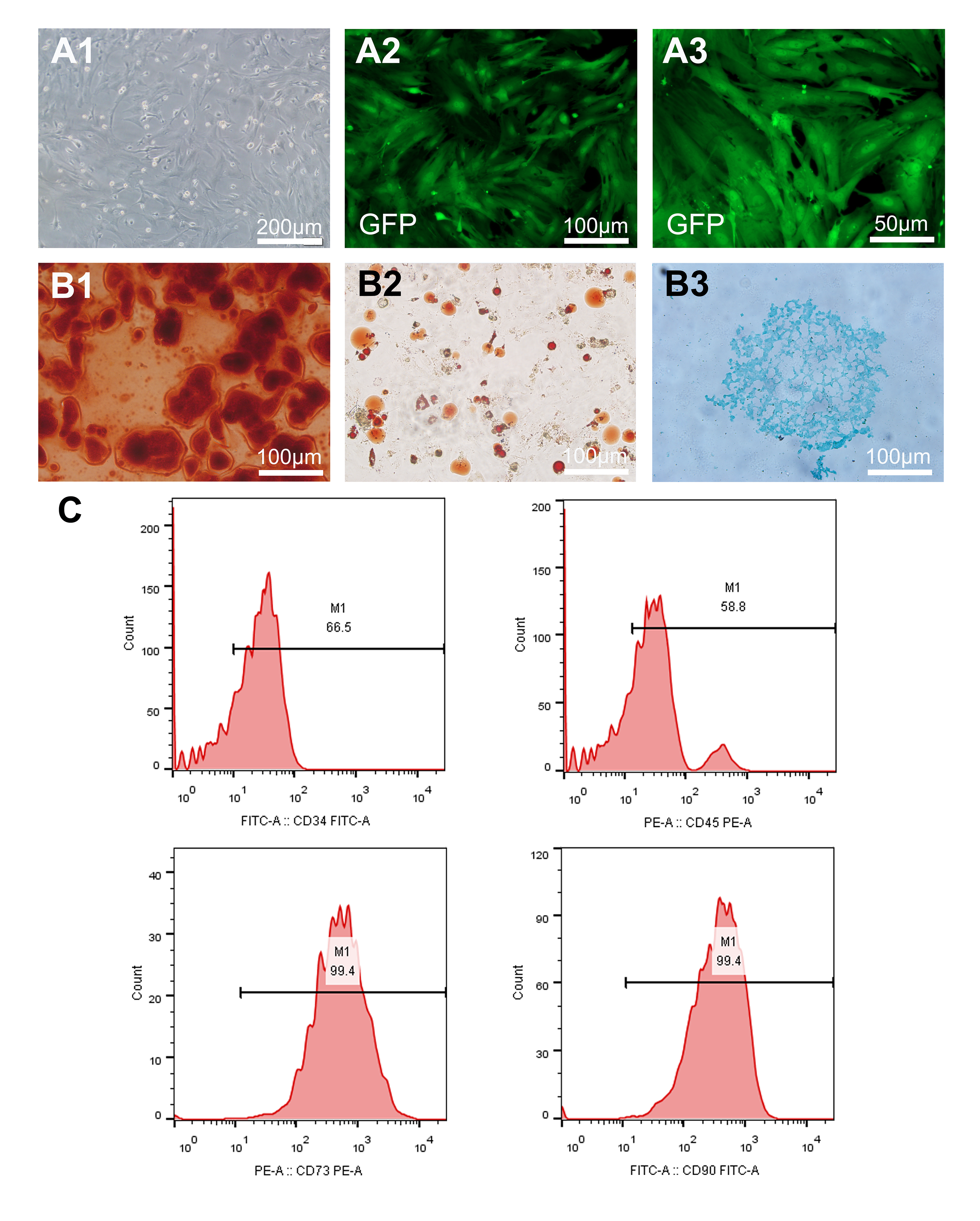


**Fig. S2.** Culture and Identification of BMSCs. (A1-A3) Primary BMSCs were spindle-shaped and expressed green fluorescence. (B1-B3) BMSCs were positive for alizarin red, oil red O, and alcian blue staining after induced differentiation. (C) Low expression of CD-34 and CD-45 and high expression of CD-73 and CD-90, the surface markers of BMSCs.


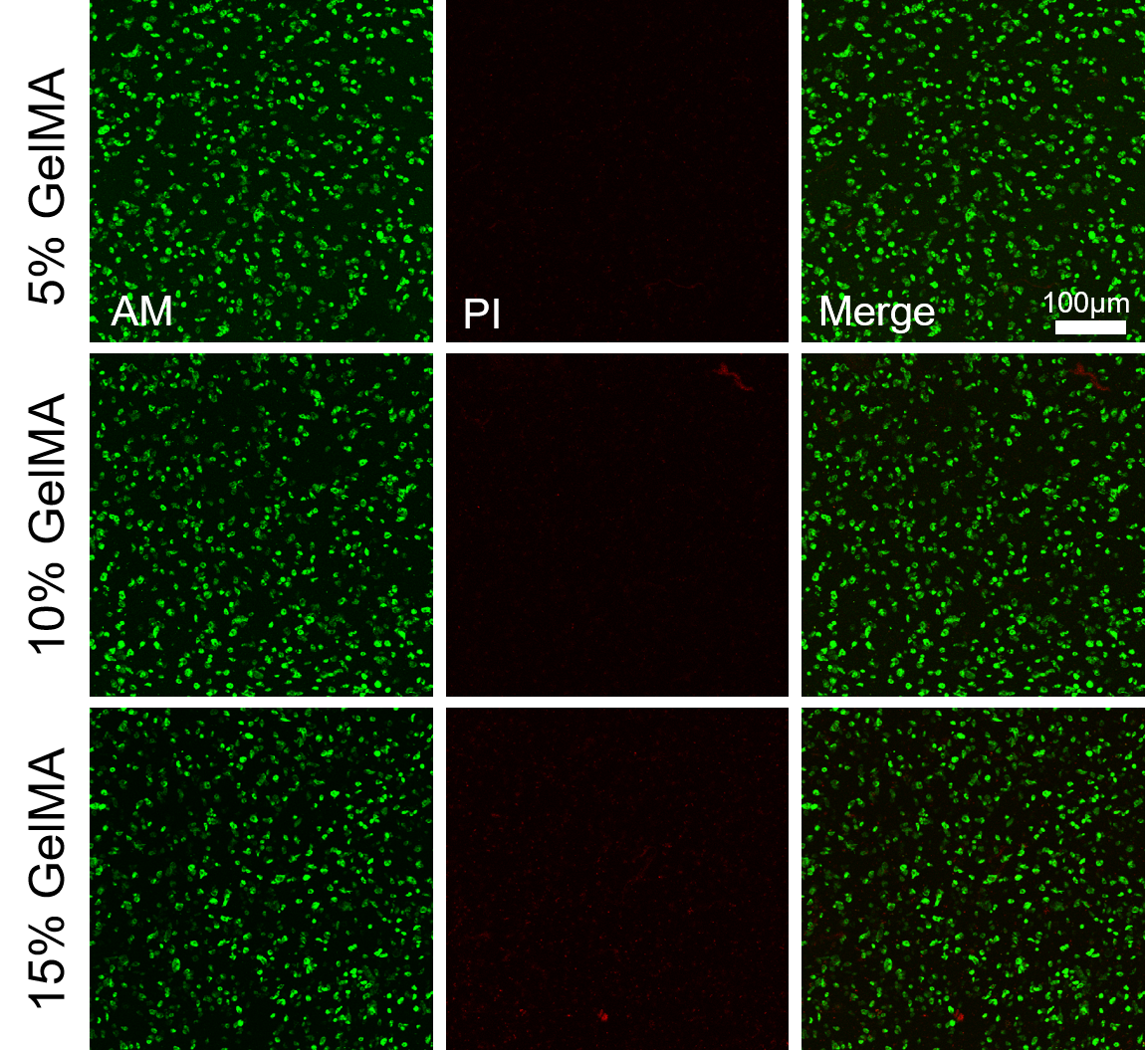


**Fig. S3.** The images of live cells, dead cells, and their staining.


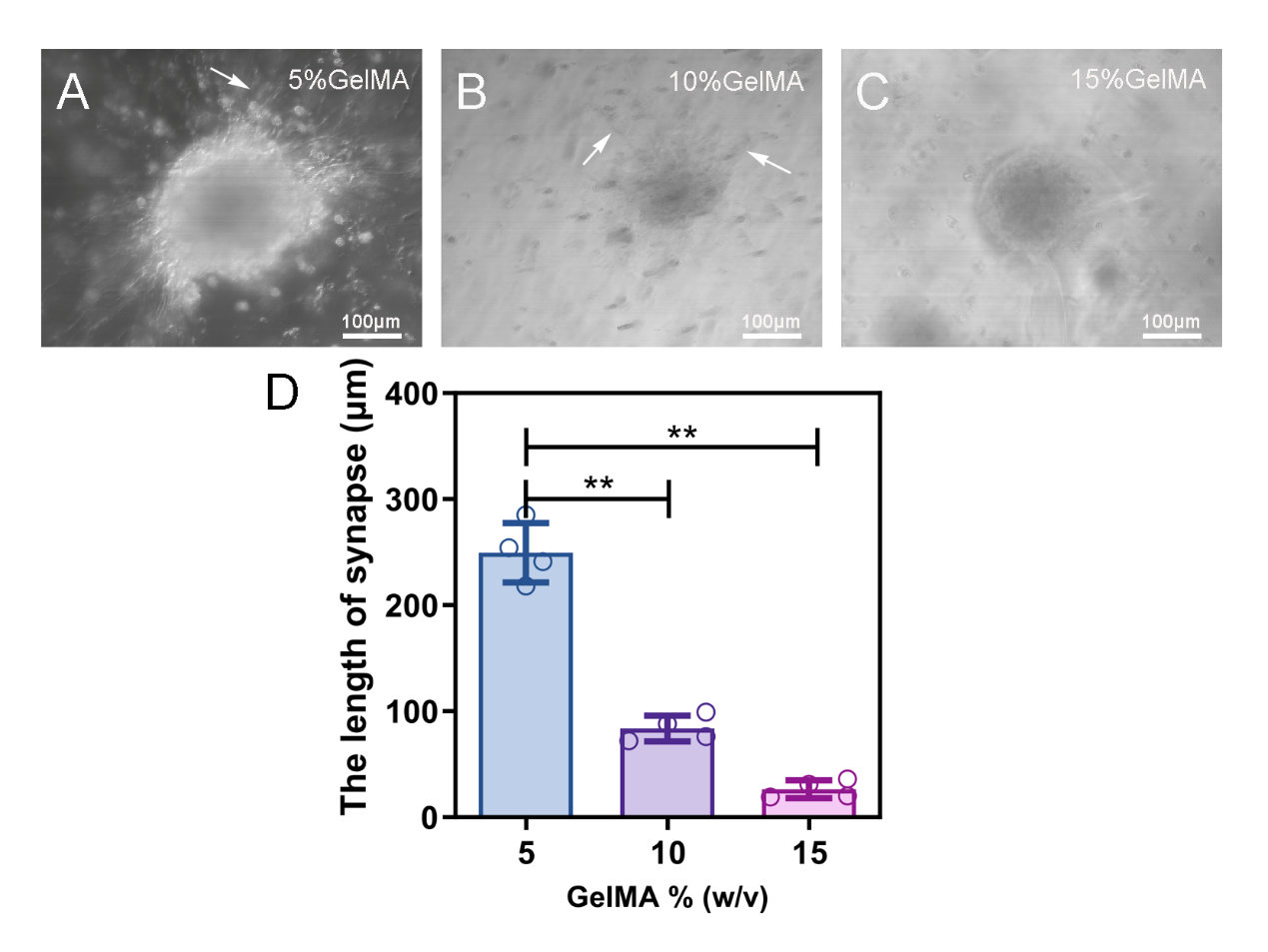


**Fig. S4.** Behavioral characterization of cells encapsulated in GelMA hydrogels. (A-C) LSCM was used to observe the morphology of cells encapsulated in the three hydrogels. (D) Neurite length statistics. (n = 3; **p < 0.01)


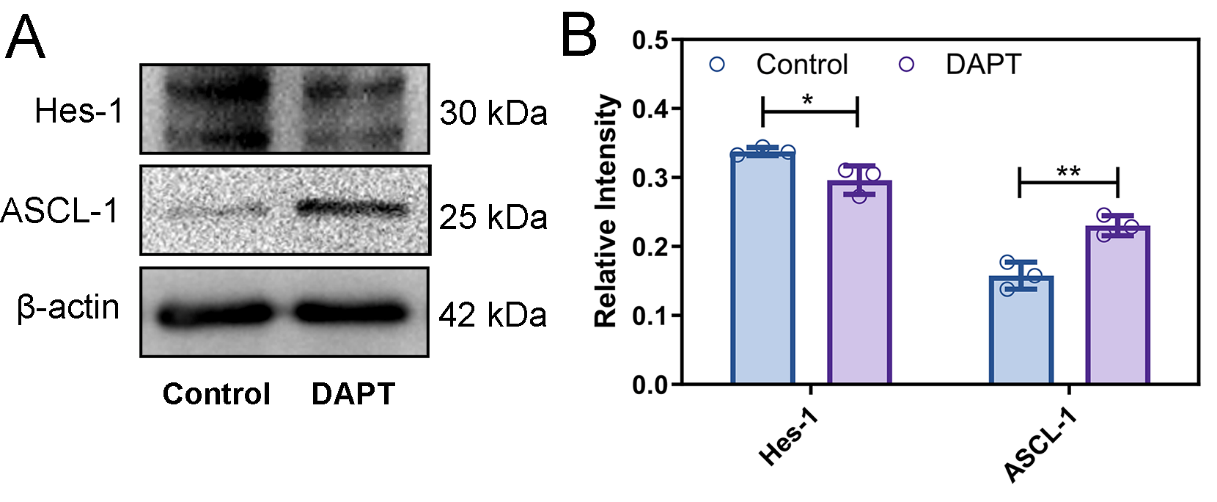


**Fig. S5.** BMSCs influence the differentiation and proliferation of NSCs through the Notch pathway. (A-B) Expression and quantification of Notch signaling pathway-related proteinsHes-1 and ASCL-1. (n = 3; *p < 0.05, **p < 0.01)
